# Effects of Steel Slag and Ethylenediaminetetraacetic Acid (EDTA) on Enhancing the CO_2_ Sequestration Performance of Gangue-Based Cemented Backfill Materials

**DOI:** 10.3390/ma18214852

**Published:** 2025-10-23

**Authors:** Xinying Li, Dan Kang, Zejun Li, Nan Zhou, Qian Chen

**Affiliations:** 1 Key Laboratory of Deep Coal Resource Mining of Ministry of Education, School of Mines, China University of Mining and Technology, Xuzhou 221116, China; lixinying@cumt.edu.cn (X.L.); zhounanyou@126.com (N.Z.); 13903977107@163.com (Q.C.); 2 Yankuang Energy Group Company Limited, Jining 273500, China; yan_kd@163.com; 3 Jiangsu Key Laboratory for Clean Utilization of Carbon Resources, China University of Mining and Technology, Xuzhou 221116, China

**Keywords:** cemented paste backfill, CO_2_ sequestration performance, steel slag, strength, CO_2_ sequestration experiment

## Abstract

**Highlights:**

**What are the main findings?**

- Proper steel slag and EDTA improved cemented paste backfill strength and CO_2_ uptake.
- Strength and CO_2_ uptake rose then fell with slag/EDTA, optimum at 10% slag, 0.5 g/L EDTA.
- EDTA chelates Ca^2+^/Mg^2+^ in steel slag, enhancing ion release and catalyzing carbonation.
- CO_2_ sequestration consumes hydration products, reducing compressive strength of backfill.

**What are the implications of the main findings?**

- Synergistic steel slag and EDTA optimize backfill by balancing strength and CO_2_ uptake.
- The results provide a feasible pathway for the co-utilization of coal-based wastes and steel slag.
- This approach advances green mining and high-value steel slag use.

**Abstract:**

To enhance the support capacity of cemented paste backfill (CPB) in goaf areas and its ability to sequester CO_2_, steel slag and ethylenediaminetetraacetic acid (EDTA) were incorporated into gangue-based cemented backfill materials. A stress–carbonation-coupled reaction system was employed to carbonate the CPB, and the effects of steel slag and EDTA on compressive strength, CO_2_ uptake, and microstructure were studied. The findings indicate that steel slag remarkably enhanced the performance of the CPB, with both strength and CO_2_ uptake initially increasing before declining as steel slag content increased. The optimum performance was achieved at a steel slag content of 10%. The incorporation of EDTA further enhanced the compressive strength and CO_2_ uptake, with the best results at 0.5 g/L. Microstructural analyses demonstrated that steel slag increased the availability of Ca^2+^ and Mg^2+^ in the cement paste system, while EDTA accelerated their leaching, promoted hydration products, and catalyzed carbonation via chelation. However, excessive steel slag or EDTA reduced hydration products and deteriorated material performance. This work may provide a reference for enhancing the properties of CPB and promoting the efficient utilization of coal-based solid wastes.

## 1. Introduction

Coal has long served as a cornerstone energy source, underpinning national economic growth and social development. However, vast underground goaf areas have been generated by extensive coal mining. In 2024, global raw coal production amounted to approximately 9.24 billion tons, with China’s output reaching 4.78 billion tons, resulting in more than 3 billion m^3^ of underground void space [1,2,3,4]. These goafs pose serious environmental and safety risks, including spontaneous combustion of residual coal, water accumulation, ground subsidence, and damage to the aquifer. Cemented paste backfill (CPB) mining technology has emerged as an effective approach to mitigate these issues. By utilizing waste materials derived from coal, including fly ash and coal gangue, in combination with a cementitious binder, CPB is injected into the goafs to stabilize the overlying strata while achieving solid waste recycling [5,6,7,8]. At the same time, the substantial CO_2_ emissions associated with coal production and utilization remain a critical environmental concern [9,10,11]. Carbon capture, utilization, and storage (CCUS) technologies provide a promising pathway for reducing these emissions. Within this framework, the carbonation pathway—using CPB as a CO_2_ absorption medium—offers a promising approach for permanent geological storage of CO_2_, thereby enabling long-term geological storage of CO_2_ [12,13].

Furthermore, the massive discharge of steel slag has led to severe soil and air pollution [14,15]. Notably, mineral phases identified in steel slag include dicalcium silicate (C_2_S), tricalcium silicate (C_3_S), and dicalcium ferrite (C_2_F) [16,17,18], which are similar to those found in cement clinker and possess potential cementitious activity [19,20,21]. Consequently, steel slag substituting for cement in backfill materials both facilitates the utilization of steel slag and reduces the environmental burden of direct disposal [22,23,24]. In recent years, considerable research efforts have focused on this topic. Li et al. [25] prepared high-carbonation precast concrete by partially replacing cement with steel slag. Their study found that under the optimal mix proportion—with a water-to-binder ratio of 0.18, 8% silica fume, and 40% sand content—replacing 80% of cement with steel slag resulted in a concrete compressive strength of 104.9 MPa, significantly enhancing its mechanical performance. Rosales et al. [26] systematically investigated the effects of different carbonation conditions on products using steel slag as raw material. The study found that the degree of carbonation significantly increased with rising temperature and CO_2_ concentration, resulting in well-crystallized carbonation products and a denser pore structure. This effectively achieved CO_2_ sequestration and improved the reactivity of steel slag, providing a basis for its resource utilization in re-cemented materials. Herki et al. [27] experimentally analyzed the influence of different steel slag incorporation rates on the microstructure and hydration products of cement paste. The results showed that an appropriate amount of steel slag promoted the formation of C-S-H gel and Ca(OH)_2_, thereby improving structural density and compressive strength. When steel slag replaced 30% of cement, the compressive strength increased by approximately 45% compared to the reference group. However, excessive incorporation led to an increase in free CaO and porosity, resulting in a decline in material performance. Similarly, Gu et al. [28] studied the effects of composite activators in a steel slag–slag cementitious system. reporting that the system achieved the highest compressive strength at a 20% steel slag content, whereas higher contents (>40%) led to strength reduction.

However, previous studies have revealed that steel slag exhibits significant volumetric instability [29,30]. Excessive incorporation of steel slag might cause material expansion, thereby restricting its large-scale utilization in engineering practice [31,32]. To address this limitation, researchers have investigated the use of various chemical activators to enhance the reactivity and dimensional stability of steel slag, achieving notable progress. Ethylenediaminetetraacetic acid (EDTA) is a strong chelating agent commonly used for titrating free lime [33], periclase [34], and metal ions such as Ca^2+^ and Mg^2+^ [35]. It also serves as a wastewater treatment agent for removing heavy metal ions from water. Due to its chelating capacity, EDTA can promote the release of metal ions from steel slag [36], enhance carbonation efficiency, and improve its volume stability [37,38,39]. Numerous scholars have conducted research on this topic.

Ababneh et al. [40] employed EDTA to pretreat medical waste fly ash and investigated its feasibility for application in mortar. Experimental results demonstrated that EDTA effectively removed heavy metal ions from the fly ash. The treated fly ash could replace up to 20% of cement in mortar preparation, achieving a material strength of 20 MPa after 28 days of curing. Chen et al. [41] incorporated EDTA into the wet carbonation process of steel slag and investigated its impact on mineralization efficiency and the properties of cementitious materials. Their results demonstrated that EDTA effectively improved both the volume stability and the mineralization efficiency of steel slag, enabling the cementitious materials to achieve a uniaxial compressive strength of 48 MPa after 28 days of curing. Katre et al. [42] studied the CO_2_ mineralization behavior and mineral transformation patterns of gabbro-peridotite and picritic basalt in solutions containing NaHCO_3_ and Na_2_H_2_EDTA·2H_2_O. The results indicated that EDTA enhanced carbonate formation by promoting mineral dissolution and the release of Ca^2+^ and Mg^2+^, significantly accelerating the mineralization rate. Thumm et al. [43] investigated the influence of EDTA on the adsorption behavior of Eu (III) and Cm (III) on calcium silicate hydrate under high-salinity conditions and varying calcium-to-silicon ratios. The findings revealed that under high-calcium conditions, stable ternary Ca–An (III)/Ln (III)–EDTA complexes formed, effectively stabilizing An^3+^ and Ln^3+^ in the liquid phase and inhibiting their incorporation into the C-S-H structure. This significantly enhanced their mobility, confirming the catalytic role of EDTA in the leaching of metal ions from steel slag. Similarly, Yichao Zhang et al. [44] examined the EDTA-assisted semi-dry carbonation process and found that EDTA markedly enhanced CO_2_ sequestration performance by chelating Ca^2+^, promoted cement hydration, and increased the strength of mortars incorporating carbonated steel slag. Nevertheless, existing research has primarily focused on the effects of EDTA on steel slag, with relatively few studies investigating the combination of steel slag and EDTA in cemented paste backfill (CPB) systems.

Based on the above research, this study integrates steel slag into CPB to systematically evaluate the effect of slag content on the mechanical performance and CO_2_ sequestration behavior of the backfill. Following the identification of the optimal steel slag content, EDTA was introduced as a chelating regulator to further enhance the performance of the CPB. The synergistic influences of steel slag and EDTA on the compressive strength and CO_2_ sequestration performance of CPB were comprehensively investigated. Furthermore, X-ray diffraction (XRD), scanning electron microscopy, and energy-dispersive X-ray spectroscopy (SEM–EDS), and other microstructural characterization techniques were employed to reveal the underlying mechanisms governing the effects of steel slag and EDTA on the microstructure and performance of the CPB.

## 2. Materials and Methods

### 2.1. Raw Materials

**Figure 1:**
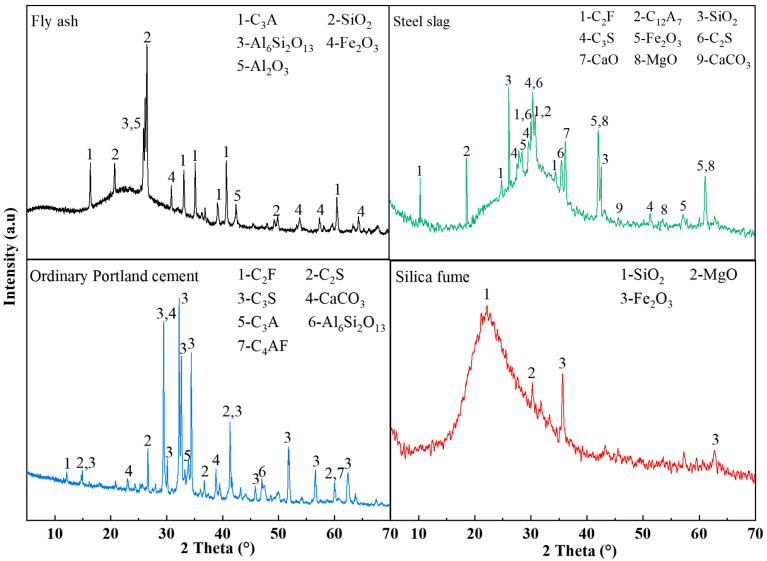
XRD patterns of raw materials.

**Figure 2:**
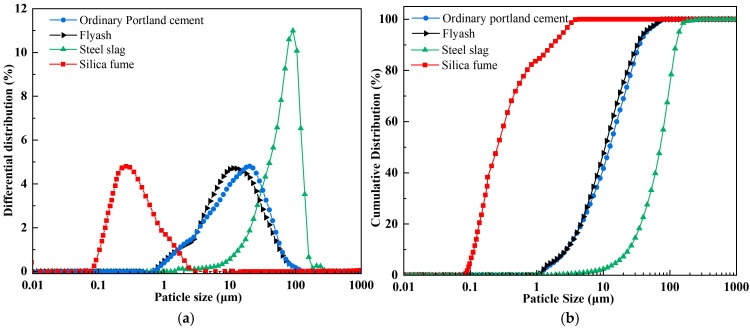
Particle size distribution of raw materials: (**a**) differential distribution; (**b**) cumulative distribution.

The raw materials utilized in this investigation encompassed coal gangue (CG), fly ash (FA), ordinary Portland cement (OPC), steel slag (SS), silica fume (SF), and ethylenediaminetetraacetic acid (EDTA). Chemical compositions of these materials were characterized using X-ray fluorescence (XRF); the detailed analytical data are listed in Table 1. The coal gangue was sourced from the Ordos coal-producing region of Inner Mongolia, with particle sizes of 0–2 mm: 2–5 mm: 5–10 mm in a mass ratio of 1:1:1. Both the fly ash and steel slag were supplied by Henan Borun Foundry Materials Co., Ltd. The mineral phase of the materials was characterized by XRD, and the principal mineral phase identified as corundum (Al_2_O_3_), while the main mineral phase of the steel slag was calcium oxide (CaO). The OPC used was P.O.42.5 ordinary Portland cement from China Huai’an Conch Cement Co., Ltd., with dominant mineral phases being CaO and quartz (SiO_2_). The main mineral phase of the silica fume was SiO_2_. The EDTA used was of analytical grade and obtained from China Tianjin Zhonglian Chemical Reagent Co., Ltd. The mineralogical compositions of the FA, SS, OPC, and SF, characterized by XRD, are presented in Figure 1. The median particle sizes (*D*_50_) of SF, SS, FA, and OPC were 0.24 μm, 71.82 μm, 10.60 μm, and 12.37 μm, respectively. The particle size distributions of FA, SS, OPC, and SF are presented in Figure 2.

### 2.2. Specimen Preparation

Test specimens were prepared with varying steel slag contents and EDTA concentrations. Based on preliminary experiments considering both compressive strength and CO_2_ sequestration performance, the mass ratio of gangue, fly ash, and cement was set at 6:3:1, with 2.5% of the cement replaced by silica fume. A constant water–to–binder ratio of 0.50 was adopted. On this basis, different variable levels were designed. First, the influence of steel slag content on the performance of the backfill was investigated. The steel slag content levels were set at 0%, 5%, 10%, and 15%, denoted as SS_0_, SS_1_, SS_2_, and SS_3_, respectively. Among these, the 10% steel slag content demonstrated the best compressive strength and carbon sequestration performance. Therefore, in the experiments involving the addition of EDTA, the steel slag content was fixed at 10% to ensure that the effects of EDTA were evaluated under the optimal steel slag conditions. And EDTA concentrations of 0 g/L, 0.1 g/L, 0.5 g/L, and 1.0 g/L, designated as E_0_, E_1_, E_2_, and E_3_, respectively. The experimental mix proportions are presented in Table 2. The specimen codes followed the format “E_x_SS_x_,” where “E_x_SS_x_-C” denotes samples after carbonation. For instance, E_1_SS_1_–C represents the sample containing 5% steel slag and 0.1 g/L EDTA after carbonation, while E_0_SS_0_ corresponds to the control group.

**Figure 3:**
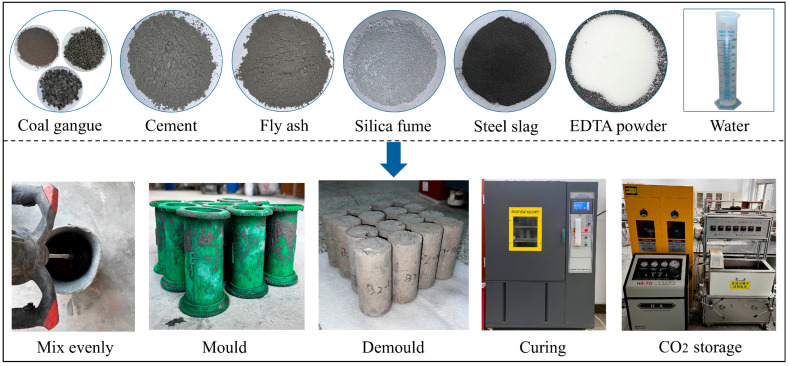
Specimen preparation procedure.

Based on the mix proportions detailed in Table 2, the solid raw materials were precisely weighed and homogenized through thorough mixing, followed by the addition of either pure water or EDTA solutions at the designated concentrations. After 5 min of uniform mixing, the prepared mixture was put into lubricated cylindrical molds (50 × 100 mm) for easy demolding. After 24 h of natural curing at ambient conditions, the samples were demolded and then transferred to a curing chamber that was maintained at 20 ± 2 °C and 95% relative humidity for curing periods of 3, 7, 14, and 28 d. The schematic diagram of the specimen preparation procedure is illustrated schematically in Figure 3**.**

### 2.3. Testing Methods

**Figure 4:**
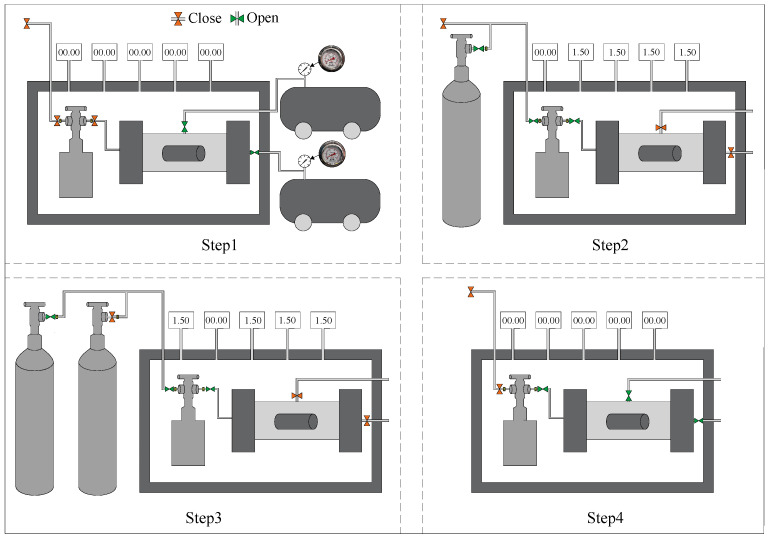
CO_2_ sequestration steps.

In this study, CO_2_ sequestration experiments were carried out using a self-developed stress–carbonation-coupled reaction vessel. The apparatus integrates triaxial stress loading, temperature regulation, and high-pressure gas injection systems, enabling simulation of the CO_2_ sequestration process of backfill materials under underground triaxial stress conditions. During testing, a confining pressure and axial pressure of 1.5 MPa were applied to the specimens, with CO_2_ continuously injected at 30 °C for a duration of 2 h. The experimental procedure for CO_2_ sequestration is illustrated in Figure 4. Following the carbonation treatment, the specimens were weighed and subsequently subjected to compressive strength testing, thermogravimetric (TG) analysis, and microstructural characterization.

#### 2.3.1. Compressive Strength

The Chinese standard GB/T 17671–2021, Methods of Testing Cements–Determination of Strength [45] was used as the basis for conducting compressive strength tests. Measurements were performed on samples at 3, 7, 14, and 28 d, as well as on specimens subjected to CO_2_ sequestration. The tests were carried out using a YAW-1000H universal testing system under displacement control at 0.5 mm/min. Test data are presented as the average of three replicates per experimental group [46].

#### 2.3.2. Carbon Sequestration Test

The CO_2_ uptake was employed to evaluate the ability of the CPB to store CO_2_. Thermogravimetric-differential thermogravimetric (TG-DTG) analysis was employed for this determination. Following CO_2_ sequestration, the specimens were ground to pass a 250-mesh sieve, and approximately 10 mg of the homogenized powder was analyzed using a simultaneous thermal analyzer METTLER TOLEDO TGA2 (Switzerland). High-purity nitrogen was used as the protective gas. The temperature was raised from ambient state to a maximum of 1000 °C, with a constant ramp rate of 10 °C/min maintained throughout the process. The carbonated CO_2_ content was quantified from the CO_2_ weight loss peaks in the TG curves. Equations (1) and (3) were applied to calculate the CO_2_ uptake and the CaCO_3_ content, respectively.

(1) $$CO_{2}uptake=\frac{CO_{2,carbonated}-CO_{2,initial}}{100-CO_{2,carbonated}}\times 100$$

(2) $$CO_{2}=\frac{\Delta m_{550{}^{\circ}C}-\Delta m_{850{}^{\circ}C}}{\Delta m_{60{}^{\circ}C}}\times 100$$

where $CO_{2,carbonated}$ denotes CO_2_ mass percentage of the carbonated sample (%); $CO_{2,initial}$ denotes initial CO_2_ mass percentage of uncarbonated sample (%); $\Delta m_{550{}^{\circ}C}$, $\Delta m_{850{}^{\circ}C}$,and $\Delta m_{60{}^{\circ}C}$ are residual mass percentage of samples at 550 °C, 850 °C and 60 °C, respectively (%).

(3) $$CaCO_{3}=CO_{2uptake}\times \frac{M_{CaCO_{3}}}{M_{CO_{2}}}$$

In the equation, $M_{CaCO_{3}}$ denotes the molar mass of CaCO_3_, and $M_{CO_{2}}$ denotes the molar mass of CO_2_ [47].

#### 2.3.3. Microstructural Analysis

XRF (Zeiss Gemini 300, Oberkochen, Germany) was used to determine the raw materials’ chemical composition. The mineralogical phases of the specimens with different mix ratios, both before and after carbonation, were identified by XRD (Ultima IV, Kyoto, Japan) equipped with Cu Kα radiation (λ = 1.541874 A, 30 kV, 40 mA). XRD analysis was performed, its scanning range was 5–70° (2θ) using a step width of 0.02° and at 4°/min. The microstructure of the samples was characterized by SEM (TESCAN MIRA4, Brno, Czech Republic) at magnifications of 500–10,000, with a resolution of 1μm. EDS was used to further characterize the elemental composition and content of the hydration products.

## 3. Results

### 3.1. Compressive Strength

**Figure 5:**
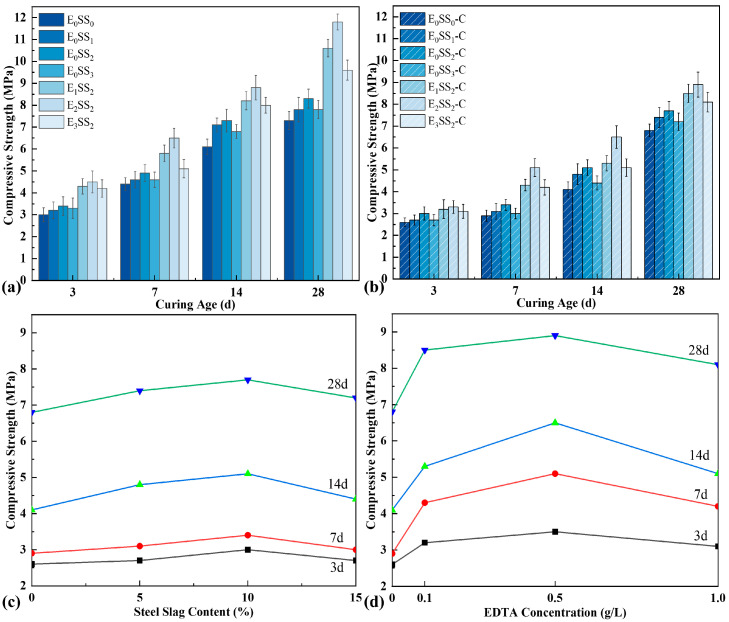
Compressive strength of specimens (**a**) before carbonation and (**b**) after carbonation; (**c**) effect of steel slag content on carbonated sample strength; (**d**) effect of EDTA concentration on carbonated sample strength.

As depicted in Figure 5, the uniaxial compressive strength of CPB at different curing ages was examined both before and after carbonation. And each data point represents the average value of three specimens. The results demonstrate that the strength of uncarbonated samples exhibited an incremental rise during the 3–7 d and 7–14 d periods, followed by a pronounced growth at 14–28 d. For the control group, the strengths were 3.0 MPa, 4.4 MPa, 6.1 MPa, and 7.3 MPa at 3, 7, 14, and 28 d, respectively. With increasing steel slag content, the compressive strength of CPB initially increased and then declined, peaking at 10% steel slag, corresponding to an improvement of 11.36–19.67% compared with the control. It is noteworthy that the early-age strength differences among specimens with varying steel slag contents were relatively minor. This may be attributed to the low early reactivity of steel slag, as the hydration of minerals such as C_2_S proceeds slowly, while the presence of a dense glassy layer on particle surfaces further restricts Ca^2+^ leaching, thereby limiting early strength development [48]. The incorporation of EDTA markedly enhanced the compressive strength. A discernible trend was observed, whereby the compressive strength initially increased and subsequently decreased with increasing EDTA concentration. Under an EDTA concentration of 0.5 g/L, the strength improvement was most pronounced, with strength increases of 44.26–61.64% over the control and 20.55–42.17% over the specimens without EDTA. When the EDTA concentration reached 1.0 g/L, the strength decreased slightly but remained higher than that of specimens without EDTA. The significant enhancement in compressive strength with EDTA addition is closely linked to its catalytic effects on hydration and carbonation reactions, which facilitate the formation of a denser and more cohesive microstructure [49].

After carbonation, the compressive strength of all specimen groups decreased to varying degrees; however, the overall trends with respect to steel slag content and EDTA concentration remained consistent with those observed before carbonation. In particular, the CPB incorporating 10% steel slag and 0.5 g/L EDTA exhibited the highest strength. At curing ages of 1–28 d, the strengths of this optimal mixture were 3.3 MPa, 5.1 MPa, 6.5 MPa, and 8.9 MPa, corresponding to an improvement of 26.92–75.86% compared with the carbonated control specimens. The reduction in strength relative to uncarbonated specimens resulted from hydration products being consumed during the carbonation process. Specifically, when CO_2_ was introduced, part of the previously formed hydration products reacted with CO_2_ to generate carbonates, thereby weakening the cementitious bonding within the matrix and resulting in a decline in compressive strength.

To evaluate the significance of the effects of steel slag content and EDTA concentration on the mechanical properties of CPB, a one-way analysis of variance (ANOVA) was performed on the compressive strength data of each group. Taking the 28 d cured specimens before carbonation as an example, the analysis results are shown in Table 3. Under the condition of only adding steel slag, a *p*-value of 0.002 was obtained, while under the condition of fixed steel slag content with the addition of EDTA, a *p*-value of <0.001 was obtained. These results indicate that both steel slag content and EDTA concentration have a significant influence on compressive strength and possess statistical significance.

### 3.2. TG-DTG

**Figure 6:**
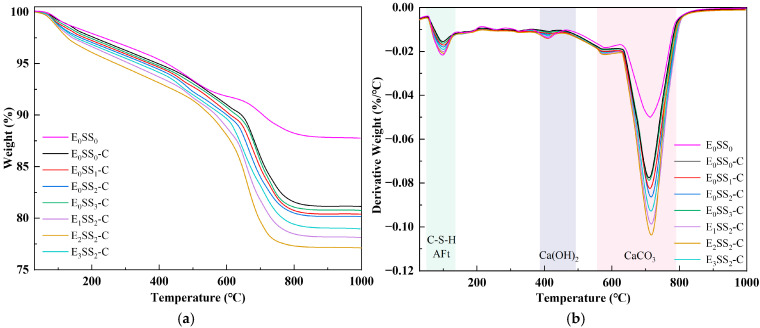
TG-DTG curves of carbonated specimens at 7 d; (**a**) TG curves; (**b**) DTG curves.

**Figure 7:**
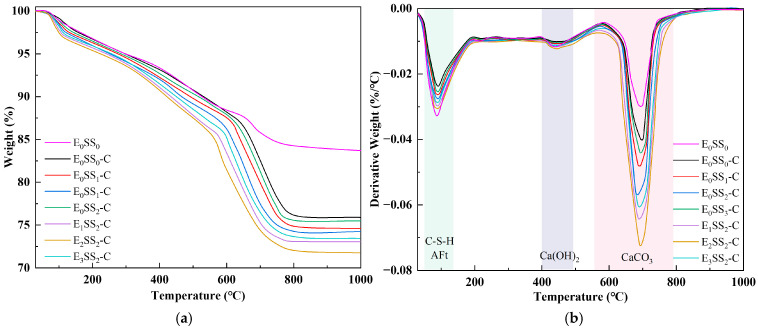
TG-DTG curves of carbonated specimens at 28 d; (**a**) TG curves; (**b**) DTG curves.

To quantify CO_2_ sequestration at different hydration and carbonation stages, TG-DTG analysis was performed on specimens cured for 7 d and 28 d. To ensure sample homogeneity and minimize experimental error, this test was performed on phenolphthalein-tested sections that remained colorless, indicating complete carbonation. Figure 6 and Figure 7 present the TG-DTG curves of the carbonated CPB after 7 d and 28 d of curing, respectively. As shown in Figure 6b and Figure 7b, three distinct weight loss peaks three distinct weight loss peaks were observed, corresponding to the dehydration of C-S-H, AFt (70–120 °C), the decomposition of Ca(OH)_2_ (400–480 °C), and CaCO_3_ (550–850 °C) were identified [50]. The TG-DTG curves indicate that, compared to the carbonated samples, the control group before carbonation exhibits larger weight loss peaks and higher weight loss rates in the temperature ranges of 0–200 °C and 400–500 °C. This suggests that the cemented backfill system before carbonation is predominantly composed of hydration products. However, in the range of 550–850 °C, the curve shows a gradual change with a slight weight loss peak, corresponding to the decomposition of a small amount of CaCO_3_. This demonstrates that the carbonate content in the samples before carbonation is relatively low, and no significant carbonation reaction has occurred in the system. Compared with the control group, the specimens containing steel slag and EDTA exhibited significantly greater weight losses, confirming the pronounced influence of steel slag content and EDTA concentration on CO_2_ uptake in the CPB. Among all specimens, those incorporating 10% steel slag and 0.5 g/L EDTA exhibited the most pronounced weight loss peaks in the 550–850 °C range at both curing ages. After 28 d of curing, the TG-DTG profiles displayed trends similar to those observed at 7 d but with lower overall weight loss. This reduction may result from the evolution of the pore structure, as continuous hydration leads to the generation of C-S-H, AFt, Ca(OH)_2_, and other products that progressively fill the pores, thereby reducing CO_2_ ingress and ultimately lowering CO_2_ sequestration capacity. These findings are consistent with the results of the compressive strength analysis.

Based on Equations (1) and (3), the CO_2_ uptake and CaCO_3_ content were calculated, and the results are summarized in Table 4. For the control group, CO_2_ uptake was 7.51% and 6.01% at 7 and 28 d, respectively, with corresponding CaCO_3_ contents of 17.15% and 13.65%. With the incorporation of steel slag and EDTA, both CO_2_ uptake and CaCO_3_ content increased. When the steel slag content increased from 0% to 15%, the CO_2_ uptake of samples at 7 d and 28 d curing ages increased by 3.14–6.79% and 1.16–3.00%, respectively, compared to the control group, while the CaCO_3_ content rose significantly. This indicates that steel slag provides additional Ca^2+^ for carbonate reaction. The maximum CO_2_ uptake of 8.02% and 6.19% was achieved at a 10% steel slag content. Building upon this optimal steel slag content, the introduction of EDTA further enhanced both CO_2_ uptake and CaCO_3_ content. Compared to the control group, the addition of 0.1 g/L to 1.0 g/L EDTA increased CO_2_ uptake by 7.46–11.05% at 7 days and 7.82–17.80% at 28 days. The sample with 0.5 g/L EDTA exhibited the highest CO_2_ uptake, reaching 8.34% and 7.09%, respectively. However, further increasing the EDTA concentration led to a decline in both CO_2_ uptake and CaCO_3_ content, which is hypothesized to result from excessive chelation of ions by EDTA, interfering with the carbonation reaction. The specific mechanism will be substantiated in the Discussion section. Additionally, the lower CO_2_ uptake at 28 days compared to 7 days is primarily attributed to the formation of more hydration products over the extended curing period, which filled pore spaces, reduced porosity, and consequently limited CO_2_ ingress into the samples. Thus, longer curing ages diminish the CO_2_ sequestration capacity of the material, a finding that will be corroborated by SEM and XRD analysis.

### 3.3. XRD

**Figure 8:**
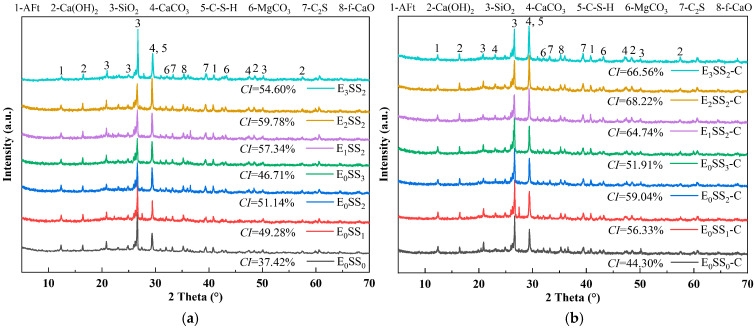
XRD patterns of samples at 28 d (**a**) before carbonation; (**b**) after carbonation.

Figure 8 presents the XRD patterns of CPB specimens with varying steel slag contents and EDTA concentrations before and after carbonation at 28 d of curing. The major diffraction peaks were identified as calcium silicate hydrate (C-S-H), ettringite (AFt), SiO_2_, and calcite (CaCO_3_). Before carbonation, the hydration products were primarily composed of C-S-H gel, accompanied by residual unhydrated C_3_S, which served as the principal contributor to the mechanical strength of the CPB [51]. C-S-H is typically amorphous or weakly crystalline, producing a broad peak near 2θ = 29.35°, while calcium hydroxide (Ca(OH)_2_) displayed characteristic peaks at 2θ = 16.47°, 48.59°, and 57.49°. In the control group, the weaker C-S-H and Ca(OH)_2_ diffraction peaks indicated a lower Ca^2+^ content in the hydration products of specimens without steel slag and EDTA [52]. After carbonation, CaCO_3_ emerged as the predominant product, with characteristic calcite peaks observed at 2θ = 29.35°, 43.18°, and 47.14°. A marked increase in the intensity of CaCO_3_ peaks was accompanied by a notable reduction in Ca(OH)_2_ and AFt. These results indicate that CaCO_3_ was primarily formed through the carbonation reaction of C-S-H gel and Ca(OH)_2_ [53]. This transformation further corroborates that the reduction in hydration products after carbonation led to a decline in the compressive strength of the CPB.

**Figure 9:**
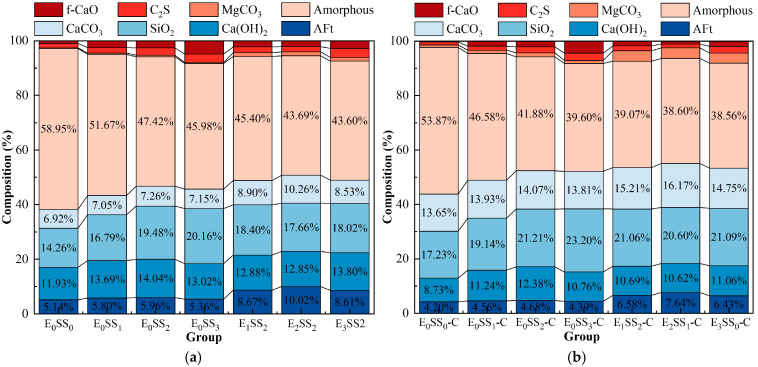
QXRD results of samples at 28 d (**a**) before carbonation; (**b**) after carbonation.

The quantitative analysis of the main mineral phases in the samples was conducted using XRD, and the Quantitative X-ray Diffraction (QXRD) results are shown in Figure 9. The incorporation of steel slag significantly altered the phase composition and reaction pathways of the system. As the steel slag content increased, the SiO_2_ content rose from 14.26% to 20.16%, and the Ca(OH)_2_ content increased from 11.93% to 14.04%. After carbonation, the Ca(OH)_2_ content decreased, while the contents of CaCO_3_ and magnesite (MgCO_3_) increased, indicating that CaO and MgO from the steel slag participated in the carbonation reaction to form CaCO_3_ and MgCO_3_. Upon the addition of EDTA, the contents of AFt and CaCO_3_ increased significantly. The highest CaCO_3_ content was observed in the sample with 10% steel slag and 0.5 g/L EDTA, which was 18.46% higher than that of the control group, demonstrating the optimal carbonation performance of this mixture and confirming that EDTA catalyzed both the hydration and carbonation reactions. It is noteworthy that the CaCO_3_ peak intensity decreased in the 15% steel slag group compared with the 10% group, and in the 1.0 g/L EDTA group compared with the 0.5 g/L group. These XRD results are consistent with the observed variations in compressive strength and CO_2_ sequestration performance of the CPB with different mix proportions.

The crystallinity index (*CI*) of each sample group was calculated from the XRD data using the peak area method [54], as shown in Figure 8. The results indicate that the crystallinity of the samples before carbonation ranged from 37.42% to 59.78%, which increased to 44.30–68.22% after carbonation. The E_2_SS_2_ group exhibited the highest crystallinity index, with a post-carbonation *CI* of 68.22%. This demonstrates that the synergistic effect of an appropriate amount of steel slag and EDTA promotes the formation of carbonate crystals, resulting in a more ordered crystal arrangement, improved structural stability, and a significant increase in crystallinity. In contrast, excessive incorporation led to a decline in crystallinity. Overall, the trend in crystallinity changes aligns with the patterns observed in compressive strength and CO_2_ sequestration: samples with higher crystallinity demonstrate superior mechanical properties and carbon sequestration capacity.

### 3.4. SEM-EDS

**Figure 10:**
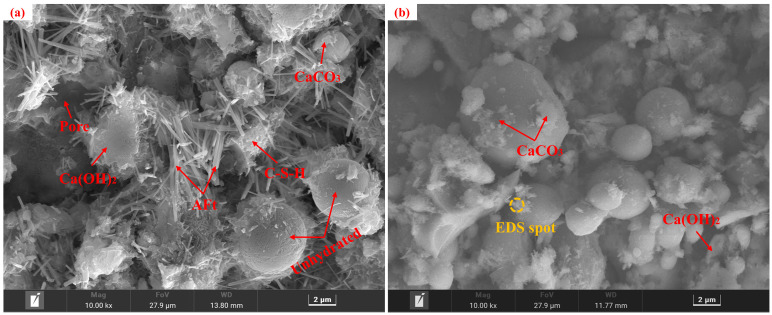

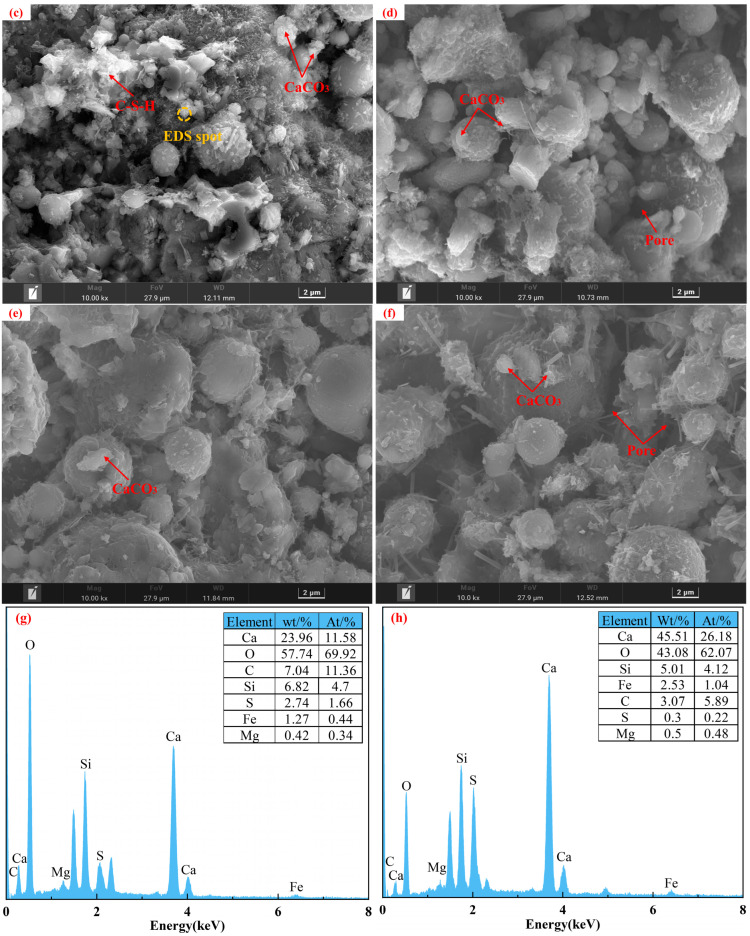
Microstructural morphology of different specimens: (**a**) E_0_SS_0_ group; (**b**) E_0_SS_0_-C group; (**c**) E_0_SS_2_-C group; (**d**) E_0_SS_3_-C group; (**e**) E_2_SS_2_-C group; (**f**) E_2_SS_3_-C group; (**g**) EDS of E_0_SS_0_-C; (**h**) EDS of E_2_SS_2_-C.

Specimens from the control group before and after carbonation at 28 d of curing, along with the carbonated E_0_SS_2_, E_0_SS_3_, E_2_SS_2_, and E_3_SS_2_ groups, were selected for SEM-EDS analysis, as shown in Figure 10. In the uncarbonated control sample, Ca(OH)_2_ crystals, unhydrated particles, and flocculent C-S-H gel were identified, exhibiting a relatively loose and porous structure [51]. After carbonation, the formation of CaCO_3_ crystals was evident, while residual Ca(OH)_2_ and C-S-H gel remained detectable. EDS analysis indicates that the primary elements on the sample surface are Ca, Si, C, O, and Mg. With the incorporation of steel slag, Mg was introduced. SEM examination revealed that the crystalline morphology of CaCO_3_ gradually evolved from regular forms to cauliflower-like forms. This transformation is attributed to Mg^2+^ promoting the growth of calcite crystals in asymmetrical directions, thereby inducing morphological changes in the crystals. This observation was corroborated by XRD mineral composition analysis [55,56]. With the incorporation of appropriate amounts of steel slag and EDTA, the generation of CaCO_3_ was further enhanced in the carbonation reaction, effectively filling pore spaces and resulting in a denser microstructure [57,58,59,60]. Furthermore, the synergy between steel slag and EDTA facilitated the formation of additional hydration products, providing a microstructural explanation for the observed improvements in the macroscopic strength of the CPB.

Excessive incorporation of steel slag or EDTA was found to affect the microstructural development of the CPB negatively. Hydration products were markedly reduced in the 15% steel slag group and the 1.0 g/L EDTA group, with visible pores within the matrix. With increasing steel slag content, the proportion of free CaO and MgO in the system increased, promoting the rapid generation of loose hydration products. In addition, the newly formed carbonates tended to envelop C-S-H gel and other hydration products, thereby impeding further carbonation reactions and gradually deteriorating the matrix structure. Similarly, at excessively high EDTA concentrations, the resulting carbonate crystals exhibited irregular sizes and indistinct morphologies. These observations were consistent with the CO_2_ sequestration results derived from TG analysis, further verifying the existence of an optimal dosage range for both steel slag and EDTA.

## 4. Discussion

### 4.1. Mechanism of Steel Slag Affecting CPB Properties

**Figure 11:**
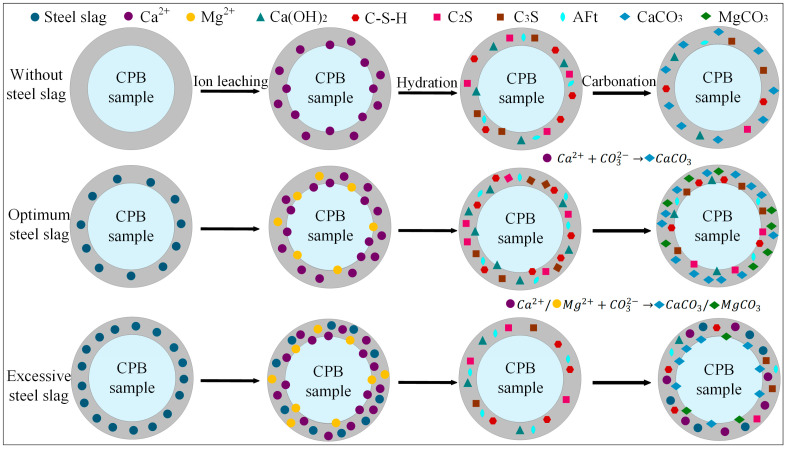
The effect mechanism of steel slag in CPB.

The results demonstrate that the incorporation of steel slag into the CPB significantly influences both its compressive strength and CO_2_ uptake. Figure 11 depicts the reaction mechanism of steel slag within the cemented backfill matrix, and the process can be broadly divided into three stages:

(1) Ion Leaching

Based on the QXRD analysis (Figure 9), it is evident that steel slag is rich in free CaO and MgO. The incorporation of 10% steel slag increased the f-CaO content in the system of the pre-carbonated samples by nearly threefold, and raised the Ca(OH)_2_ content to 14.04%. During the ion leaching stage, the hydrolysis of steel slag releases substantial amounts of Ca^2+^ and Mg^2+^, markedly increasing these ions within the matrix. Simultaneously, the calcium and magnesium oxides in steel slag react with water to form Ca(OH)_2_ and Mg(OH)_2_, and the resulting alkaline environment further promotes the continuous release of ions [19].

(2) Hydration Reaction

The incorporation of steel slag creates a highly alkaline environment, promoting the release of ions such as Ca^2+^, Mg^2+^, H_2_SiO_4_^2−^, and HSiO^3−^. The increased intensity of the C-S-H diffraction peak in the XRD pattern (Figure 8) indicates that mineral phases like C_2_S and C_3_S in steel slag undergo hydration reactions, facilitating the extensive formation of C-S-H gel. This gel effectively binds unhydrated steel slag particles, fly ash glassy phases, and gangue, forming a skeletal structure that enhances the compressive strength of the steel slag-incorporated groups. Specifically, the addition of 10% steel slag resulted in compressive strengths of 3.4 MPa, 4.9 MPa, 7.3 MPa, and 8.3 MPa for pre-carbonated samples at different curing ages, respectively. However, when the steel slag content is excessively high, surplus Ca^2+^ competes with silicate polymers ([SiO_4_]^4−^) for binding sites, leading to a reduction in C-S-H peak intensity and diminished formation of hydration products. This weakens the cementing effect within the system, a finding consistent with the research of Hao et al. [61].

(3) Carbonation Reaction

Based on the CO_2_ sequestration capacity (Table 4) and QXRD phase content results (Figure 9), it was observed that at 7 d curing age, the CO_2_ uptake could be increased by up to 6.79% compared to the control group. Under the three different steel slag incorporation rates, the CaCO_3_ content after carbonation reached 13.93%, 14.07%, and 13.81%, respectively, while the content of hydration products such as C_2_S and AFt decreased to varying degrees. Additionally, the compressive strength of the samples after carbonation was reduced by up to 54.5% compared to that before carbonation (Figure 5). CO_2_ diffuses through the pore structure and dissolves in the water, forming CO_3_^2−^, which subsequently reacts with Ca^2+^ and Mg^2+^ to generate stable carbonate minerals such as CaCO_3_ and MgCO_3_. This process enables long-term CO_2_ sequestration. However, carbonation also consumes part of the hydration products, resulting in a decrease in compressive strength after CO_2_ treatment. At higher steel slag contents, the increased concentrations of Ca^2+^ and Mg^2+^ accelerate the precipitation of carbonate crystals. These newly formed CaCO_3_ and MgCO_3_ crystals encapsulate unreacted particles, obstructing the further release of ions, impeding subsequent mineralization reactions, and ultimately diminishing the CO_2_ sequestration efficiency of the material. The mechanism is consistent with the conclusions drawn by Ababneh et al. [40].

### 4.2. Mechanism of the Synergistic Effect of Steel Slag and EDTA on CPB Properties

**Figure 12:**
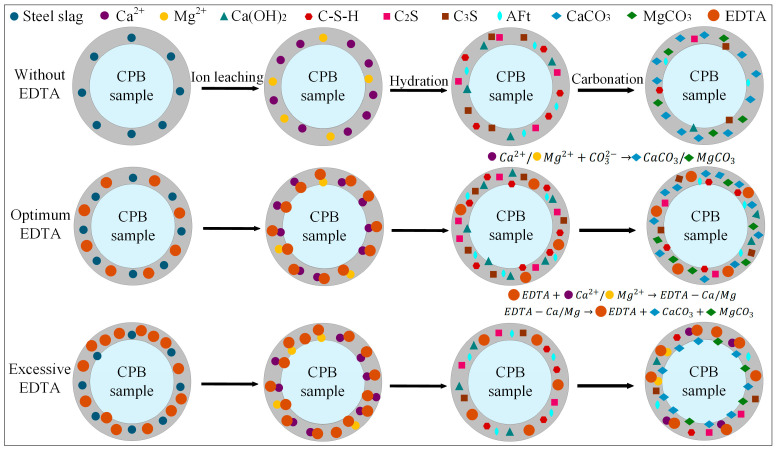
The effect mechanism of EDTA in CPB.

Based on the optimal steel slag content, the incorporation of an appropriate amount of EDTA significantly enhanced both the compressive strength and carbon sequestration capacity of the CPB. The E_2_SS_2_-C group achieved the peak compressive strength, reaching 8.9 MPa at 28 d of curing (Figure 5). This improvement is primarily attributed to the chelating effect of EDTA, with the reaction mechanism illustrated in Figure 12. Upon introduction into the system, EDTA dissolves in water and releases H^+^, promoting the leaching of calcium and magnesium ions from the steel slag [62,63]. Subsequently, EDTA complexes with Ca^2+^ and Mg^2+^ to form EDTA-Ca and EDTA-Mg, which facilitates the formation of hydration products and enhances the cementitious action of the system, thereby improving the material’s strength. As a result, the 28-day compressive strength of the samples increased by 23.08–51.28% compared to the group without EDTA. However, when the EDTA concentration was excessively high, the AFt content decreased from 7.64–6.43%, and the 28 d compressive strength dropped from 8.9 MPa to 8.1 MPa. This is because the excessively chelated Ca^2+^ competed with silicate polymers ([SiO_4_]^4−^) for binding sites, inhibiting the formation of hydration products and weakening the cementing effect of the system. After carbonation, the contents of CaCO_3_ and MgCO_3_ in the system increased by 6.81–17.09% and 235.71–247.32%, respectively, compared to the group without EDTA (Figure 9), leading to a corresponding improvement in CO_2_ sequestration capacity. During the carbonation process, the decomposition of EDTA-Ca and EDTA-Mg complexes promotes the precipitation of carbonate compounds, primarily CaCO_3_ and MgCO_3_, thereby enhancing CO_2_ uptake and improving carbonation efficiency. However, at high EDTA concentrations, the accelerated precipitation of CaCO_3_ and MgCO_3_ results in carbonate layers that coat unreacted particles, hindering further ion leaching. This reduces the availability of Ca^2+^ and Mg^2+^ for subsequent mineralization [24], ultimately limiting the carbonation efficiency—an effect similar to that observed with excessive steel slag incorporation. Therefore, appropriate dosages of both EDTA and steel slag are crucial for maximizing the strength and CO_2_ sequestration performance of CPB, whereas overdosing diminishes these benefits. This finding aligns with the results reported by Chen et al. [41].

## 5. Conclusions

This study investigated the effects of steel slag content and EDTA concentration on compressive strength and CO_2_ sequestration performance of CPB. It further analyzes the mechanisms by which steel slag, and the synergistic interaction between steel slag and EDTA, influence material performance. The main conclusions can be drawn:

(1) Compared with the control group, the incorporation of appropriate incorporation of steel slag and EDTA significantly enhanced the compressive strength of the CPB. With increasing steel slag content and EDTA concentration, the compressive strength first increased and then decreased, with the optimal mixture containing 10% steel slag and 0.5 g/L EDTA. Under these conditions, the specimen achieved a compressive strength of 6.4 MPa after 28 d of curing.
(2) The incorporation of steel slag and EDTA also significantly improved the CO_2_ sequestration performance of the CPB, exhibiting a similar trend to that of compressive strength with an optimal dosage. Under the optimal conditions of 10% steel slag and 0.5 g/L EDTA, the specimen cured for 7 d achieved a CO_2_ uptake of 7.94%. Moreover, extending the curing age reduced the CO_2_ uptake, as the value after 28 d of curing was lower than that after 7 d.
(3) The incorporation of steel slag and EDTA affects the performance of cemented backfill materials across the three crucial stages of ion leaching, hydration, and carbonation. Steel slag serves as a source for additional Ca^2+^ and Mg^2+^ ions, while the presence of EDTA significantly enhances their leaching and dissolution into the aqueous phase. This synergistic action accelerates the formation of both hydration products and carbonates within the cemented matrix, leading to a stable skeletal structure, improved compressive strength, and enabling long-term CO_2_ sequestration.
(4) Conversely, when the content of steel slag or the concentration of EDTA is excessively high, a detrimental effect is observed. The resulting surplus of Ca^2+^ and Mg^2+^ ions leads to competitive binding with silicate polymers ([SiO_4_]^4−^), thereby reducing the formation of hydration products. Furthermore, excessive carbonate precipitates may rapidly form and fully envelop the unreacted particles and existing hydration products, restricting further reaction processes. This ultimately creates porosity and matrix deterioration, leading to a decline in both the material’s compressive strength and carbonation performance.

## Figures and Tables

**Table 1:** Chemical composition of raw materials.

Materials	CaO (%)	Al_2_O_3_ (%)	SiO_2_ (%)	SO_3_ (%)	Fe_2_O_3_ (%)	P_2_O_5_ (%)	MnO (%)	MgO (%)	TiO_2_ (%)	K_2_O (%)	Na_2_O (%)
FA ^1^	4.50	36.80	45.10	1.20	3.90	/	/	1.86	0.12	1.11	2.75
SS ^2^	38.72	6.37	19.40	0.81	22.42	1.38	2.10	6.05	0.88	/	/
OPC ^3^	54.36	9.76	22.31	3.16	3.13	0.13	0.04	1.01	0.43	1.03	0.20
SF ^4^	0.62	0.53	90.27	0.36	1.66	0.07	/	2.51	/	1.34	1.34

^1^ FA is Fly ash; ^2^ SS is Steel slag; ^3^ OPC is Ordinary Portland cement; ^4^ SF is Silica fume.

**Table 2:** Mix proportions of samples.

Samples	CG ^1^(%) /(kg/m^3^)	FA ^2^(%) /(kg/m^3^)	OPC ^3^(%) /(kg/m^3^)	SF ^4^(%) /(kg/m^3^)	Water Binder	EDTA ^5^ (g/L)	SS ^6^(%) /(kg/m^3^)
E0SS0	60 /1380	30 /690	7.5 /172.5	2.5 /57.5	0.5	/	/
E0SS1						/	5/115
E0SS2						/	10/230
E0SS3						/	15/345
E1SS2						0.1	10/230
E2SS2						0.5	10/230
E3SS2						1.0	10/230

^1^ CG is Coal gangue; ^2^ FA is Fly ash; ^3^ OPC is Ordinary Portland cement; ^4^ SF is Silica fume; ^5^ EDTA is Ethylenediaminetetraacetic Acid; ^6^ SS is Steel slag.

**Table 3:** ANOVA analysis of 28 d compressive strength before carbonation.

Factor	Source of Variation	SS ^1^	df ^2^	MS ^3^	F-Value ^4^	*p*-Value ^5^
Steel Slag	Within the group	1.755	3	0.585	10.74	0.002
	Between groups	0.436	8	0.0545		
	Total	2.191	11			
EDTA	Within the group	20.295	3	6.765	124.1	<0.0001
	Between groups	0.436	8	0.545		
	Total	20.731	11			

^1^ SS is the sum of squares; ^2^ df is the degrees of freedom; ^3^ MS is the mean square; ^4^ F is the statistic; ^5^ *p* is the significance level.

**Table 4:** CO_2_ uptake and CaCO_3_ content of carbonated samples.

Curing Time (d)	Samples	CO_2_ Uptake (%)	Estimated Content of CaCO_3_ (%)
7	E_0_SS_0_-C	7.51	17.15
	E_0_SS_1_-C	7.82	17.76
	E_0_SS_2_-C	8.02	18.31
	E_0_SS_3_-C	7.77	17.75
	E_1_SS_2_-C	8.18	18.86
	E_2_SS_2_-C	8.34	19.05
	E_3_SS_2_-C	8.07	18.43
28	E_0_SS_0_-C	6.01	13.65
	E_0_SS_1_-C	6.13	13.93
	E_0_SS_2_-C	6.19	14.07
	E_0_SS_3_-C	6.08	13.81
	E_1_SS_2_-C	6.68	15.21
	E_2_SS_2_-C	7.09	16.17
	E_3_SS_2_-C	6.48	14.75

## Data Availability

The original contributions presented in this study are included in the article. Further inquiries can be directed to the corresponding author.

## Abbreviations

The following abbreviations are used in this manuscript:

EDTA	Ethylenediaminetetraacetic Acid
CPB	Cemented paste backfill
CCUS	Carbon capture, utilization, and storage
XRD	X-ray diffraction
SEM	Scanning electron microscopy
CG	Coal gangue
FA	Fly ash
OPC	Ordinary Portland cement
SS	Steel slag
SF	Silica fume
XRF	X-ray fluorescence spectroscopy
TG	Thermogravimetric
DTG	Differential thermogravimetric
QXRD	Quantitative X-ray Diffraction
CI	crystallinity index
